# Genome-Wide Analysis of *LIM* Family Genes in Foxtail Millet (*Setaria italica* L.) and Characterization of the Role of *SiWLIM2b* in Drought Tolerance

**DOI:** 10.3390/ijms20061303

**Published:** 2019-03-15

**Authors:** Rui Yang, Ming Chen, Jian-Chang Sun, Yue Yu, Dong-Hong Min, Jun Chen, Zhao-Shi Xu, Yong-Bin Zhou, You-Zhi Ma, Xiao-Hong Zhang

**Affiliations:** 1 State Key Laboratory of Crop Stress Biology for Arid Areas, College of Life Sciences, College of Agronomy, Northwest A&F University, Yangling 712100, China; Yangr1213@126.com (R.Y.); yuyue5911@163.com (Y.Y.); mdh2493@126.com (D.-H.M.); 2 Institute of Crop Science, Chinese Academy of Agricultural Sciences (CAAS)/National Key Facility for Crop Gene Resources and Genetic Improvement, Key Laboratory of Biology and Genetic Improvement of Triticeae Crops, Ministry of Agriculture, Beijing 100081, China; chenming02@caas.cn (M.C.); chenjun@caas.cn (J.C.); xuzhaoshi@caas.cn (Z.-S.X.); zhouyongbin@caas.cn (Y.-B.Z.); 3 Institute of Crop Research, Ningxia Academy of Agriculture and Forestry Sciences, Yongning 750105, China; nxsjch@163.com

**Keywords:** genome-wide analysis, drought tolerance, foxtail millet, *LIM* genes, transgenic rice

## Abstract

LIM proteins have been found to play important roles in many life activities, including the regulation of gene expression, construction of the cytoskeleton, signal transduction and metabolic regulation. Because of their important roles in many aspects of plant development, *LIM* genes have been studied in many plant species. However, the *LIM* gene family has not yet been characterized in foxtail millet. In this study, we analyzed the whole genome of foxtail millet and identified 10 *LIM* genes. All *LIM* gene promoters contain MYB and MYC *cis*-acting elements that are related to drought stress. Based on the presence of multiple abiotic stress-related *cis*-elements in the promoter of *SiWLIM2b*, we chose this gene for further study. We analyzed *SiWLIM2b* expression under abiotic stress and hormone treatments using qRT-PCR. We found that *SiWLIM2b* was induced by various abiotic stresses and hormones. Under drought conditions, transgenic rice of *SiWLIM2b*-overexpression had a higher survival rate, higher relative water content and less cell damage than wild type (WT) rice. These results indicate that overexpression of the foxtail millet *SiWLIM2b* gene enhances drought tolerance in transgenic rice, and the *SiWLIM2b* gene can potentially be used for molecular breeding of crops with increased resistance to abiotic stress.

## 1. Introduction

LIM domain proteins are widely found in eukaryotes [1] and have been found to play important roles in many aspects of development, including the regulation of gene expression, cytoskeleton assembly, signal transduction and the regulation of metabolism [2,3,4,5,6]. In animals, LIM proteins have also been found to play a role in cell adhesion and cell movement, participating in tumorigenesis and metastasis [7]. The LIM domain was first discovered in the three homeodomain proteins LIN11 (lineage 11), ISl-1 (insulin I) and MEC-3 (Mechanotransduction 3) [8,9,10,11], and was named based on the first letters of these protein names [11]. The LIM domain consists of a double zinc finger sequence, which contains two conserved sequences (C-X2-C-X16-23-H-X2-C-X2-C-X16-23-Z-X2-C) 50–60 amino acids in length that are separated by two hydrophobic amino acid residues [12].

The first LIM domain protein in plants was identified in sunflower pollen tubes [13], and to date many LIM domain proteins have been identified in plant species including tobacco [14,15], *Arabidopsis thaliana* [16,17,18], poplar [18], cotton [19,20,21], and tomato [22]. Plants have two different LIM domain protein subfamilies: CRP and DA1&DAR. Members of the CRP subfamily contain two conserved LIM domains similar to those of the cysteine-rich protein (CRP) family members in animals [18]. The two LIM domains are separated by a long inter-LIM domain. Unlike animal CRP LIM domain proteins, plant LIM domain proteins contain a short C-terminus and lack a glycine-rich region.

CRP-like LIM domain proteins are divided into two groups based on their expression patterns: WLIMs (WLIM1 and WLIM2), which are widely expressed in various tissues, and PLIMs (PLIM1 and PLIM2), which are abundantly expressed in pollen tubes [1]. Arnaud et al. [18] re-grouped the plant CRP-like LIM proteins into four subgroups based on amino acid similarity: αLIM1, βLIM1, γLIM2 and δLIM2. PLIM1 and WLIM1 are clustered in the αLIM1 subfamily, WLIM2 belongs to the γLIM subgroup and PLIM2 belongs to the δLIM subgroup. Arnaud et al. also discovered a new group of CRP LIM-like proteins that formed a single cluster in a phylogenetic tree and named this group the βLIM1 subfamily [18]. Members of the second plant LIM subfamily, the DA1&DAR subfamily, contain a conserved LIM domain, one or more ubiquitin interaction motif (UIM) domains and a conserved C-terminal region [23]. Zhao et al. classified DA1&DAR-like LIM proteins into two categories, Class I and Class II, according to the amino acid sequence. So far, the DA1&DAR LIM proteins have only been found in land plants [23].

Plant LIM proteins are mainly involved in actin cytoskeleton remodeling and the phenylpropane secondary metabolic pathway. In the phenylpropanol pathway L-phenylalanine is used as a substrate for the synthesis of many different aromatic phenolic compounds, such as coumarin, flavonoids, stilbene, hydroxy cinnamate and lignin [24,25,26]. These substances greatly enhance the tolerance of gymnosperms and angiosperms to mechanical damage such as injury or environmental stress such as drought [24]. The biological functions of LIM domain proteins are closely related to their subcellular localization [27]. LIM domain proteins have three different localization patterns: cytoplasmic localization, nuclear localization, and co-localization in the cytoplasm and nucleus [27]. Nuclear-localized LIM proteins play a major role in tissue-specific gene regulation and cell differentiation [28]. For example, in tobacco, a nuclear-localized LIM1 protein was found to activate the expression of a β-glucuronidase reporter gene under the control of the horseradish peroxidase *prxC2* promoter [29], and suppression of *LIM1* resulted in down-regulated expression of Phe ammonia lyase (*PAL*)-box genes [5]. Based on these findings, Kawaoka and Ebinuma proposed that tobacco LIM1 is a transcription factor involved in lignin synthesis. Cytoplasmic-localized LIM proteins play a major role in regulating the cytoskeleton [28]. For example, in *Arabidopsis*, six LIM domain proteins are localized at the actin cytoskeleton and have been found to bind to actin, with binding activity regulated by pH and Ca^2+^ [17]. Nuclear-cytoplasmic co-localized LIM proteins can shuttle between the cytoplasm and nucleus to regulate actin stability and the regulation of gene expression, respectively [2]. The tobacco NtWLIM2 protein is localized in the nucleus and nucleolus, where it activates the transcription of histone genes, and is also localized at the actin cytoskeleton where it can directly bind to actin fibers, leading to linkage of the actin cytoskeleton and the aggregation of fibers into actin bundles [14]. In upland cotton (*Gossypium hirsutum*), a WLIM1a protein that is localized in the cytosol and nucleus can shuttle into the nucleus when induced by the reactive oxygen species (ROS) hydrogen peroxide (H_2_O_2_). *WLIM1a* is mainly expressed during the elongation and secondary wall synthesis stages. When cotton fibers rapidly elongate, WLIM1a acts as an actin binding protein, mainly participating in active intracellular transport. When WLIM1a induced by ROS, fiber elongation is blocked, and WLIM1a enters the nucleus where it may function as a transcription factor to activate the expression of genes in the phenylpropanol biosynthesis pathway, thereby regulating secondary wall synthesis [21].

In recent years, studies have shown that *LIM* genes up-regulated expression respond to abiotic stresses, including drought, high salinity and hormones, suggesting that *LIM* genes may play a role in plant resistance to abiotic stress [22,23,30]. The functions of *LIM* family genes in cytoskeleton formation, signal transduction and the phenylpropane secondary metabolic pathway [15,21] also suggest that this family is important for abiotic stress because these processes are also important for stress responses. For example, the cytoskeleton participates in various life activities in cells and also plays a key regulatory role in the resistance of plants to various stress responses [31,32]. Plant antitoxins synthesized via the phenylpropane metabolic pathway are important for resistance to stress as well as for growth and development [33,34]. Because of their potential importance in the response to abiotic stress, *LIM* genes have been the focus of studies in many plants.

Drought stress is one of the main factors limiting global agricultural production [35]. Foxtail millet (*Setaria italica* L.) is an important food and feed crop in arid regionsof the world [36] and has strong resistance to various abiotic stresses, especially drought and low nutrition. Therefore, foxtail millet is used as a model monocotyledon crop for abiotic stress resistance research [35]. However, the mechanism of abiotic stress resistance in foxtail millet is still largely unclear [37]. Although the *LIM* gene family potentially plays an important role in abiotic stress response, this family has not yet been characterized in foxtail millet. Here we took advantage of the foxtail millet genome sequence [38,39] to identify all the members of the *LIM* gene family. Based on the complexity of LIM protein function, we identified all *LIM* genes in foxtail millet. We identified 10 *SiLIM* genes and classified these genes into two main groups. Quantitative real-time PCR (qRT-PCR) analysis showed that a *LIM*-like gene, *SiWLIM2b*, was induced by various stresses, and overexpression of *SiWLIM2b* increased the drought resistance of transgenic rice. These results provide evidence for the functions of a valuable novel *LIM*-like gene, *SiWLIM2b*, which can potentially be used for molecular breeding of resistance to abiotic stresses in graminaceous crops.

## 2. Results

### 2.1. Phylogenetic Analysis and Chromosomal Distribution of 10 LIM-Like Genes

**Figure 1:**
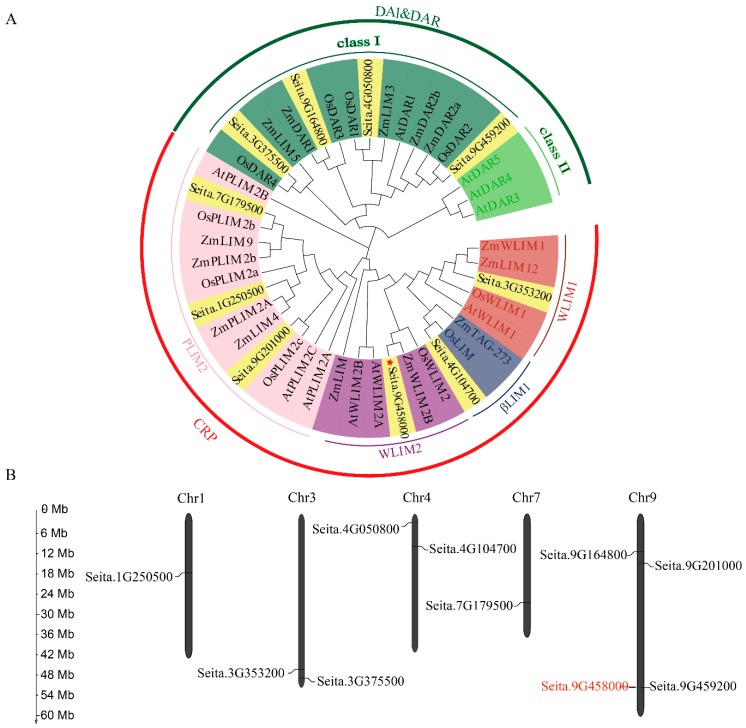
Phylogenetic analysis of foxtail millet and other plant LIM proteins and the chromosomal distribution of the 10 *SiLIM* genes. (**A**) Phylogenetic relationships of LIM family proteins in foxtail millet, *Arabidopsis*, maize and rice. The phylogenetic tree was constructed using the maximum likelihood method in Molecular Evolutionary Genetics Analysis 7.0 software based on the comparison of LIM amino acid sequences. Dark green, light green, pink, purple, dark blue and red represent the DA1&DAR class I, DA1&DAR class II, PLIM2, WLIM2, βLIM1 and WLIM1 subgroups, respectively. Yellow represents the foxtail millet *LIM* genes, and the red five-pointed star indicates the *SiWLIM2b* (*Seita.9G458000*) gene. The species acronym is shown before each LIM protein name: Seita, *Setaria italica*; At, *Arabidopsis thaliana;* Os, *Oryza sativa*; Zm, *Zea mays*. (**B**) Distribution of *SiLIM* genes in the foxtail millet genome. The *SiLIM* genes are located on chromosomes 1, 3, 4, 7 and 9. The *SiWLIM2b* gene is located on chromosome 9 and is marked in red. Chromosomal distances are given in Mb.

By searching the foxtail millet genome using the LIM domain (PF00412) as a keyword, we identifieda total of 10 putative *LIM* genes. The amino acid lengths of the LIMs ranged from 199 to 1441 residues, the predicted protein molecular weights ranged from 22.02 kDa to 154.76 kDa, and the isoelectric points ranged from 4.64 to 8.98 (Supplementary Table S1). To explore the phylogenetic relationships between the *LIM* genes, a phylogenetic tree was constructed using the LIM domain proteins from maize, rice, *Arabidopsis* and foxtail millet (Figure 1A). Phylogenetic analysis showed that foxtail millet LIMs could be divided into the DA1&DAR (four proteins) and the CPR-like (six proteins) subfamilies (Figure 1A). The DA1&DAR-like LIM proteins were further divided into Class I and Class II [23], and the CPR-like subfamily was further divided into the WLIM1, WLIM2, βLIM1 and PLIM2 subgroups (Figure 1A) [22]. We found that the *SiLIM* genes were more closely related to genes from maize and rice than to genes from *Arabidopsis* (Figure 1A).

To visualize the genome distribution of the *LIM* genes in foxtail millet, the MapGene2Chrom tool [40] was used to map the chromosomal locations. The *SiLIM*s were mapped to chromosomes 1, 3, 4, 7 and 9 (Figure 1B), with most genes located on chromosome 9 (*Seita.9G164800*, *Seita.9G201000*, *Seita.9G459200* and *Seita.9G458000*) (Figure 1B). Thus, the *LIM* genes are unevenly distributed in the foxtail millet genome. The precise locations of the 10 *LIM* genes are listed in Supplementary Table S1.

### 2.2. Functional Domains and Gene Structure Analysis of the 10 LIM-Like Genes

**Figure 2:**
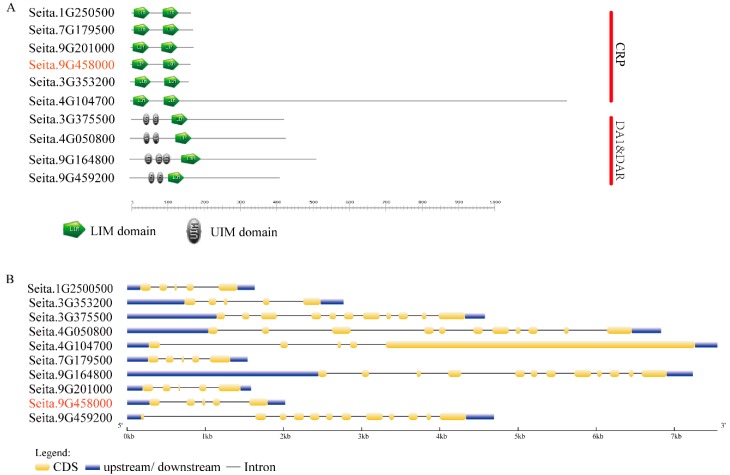
Functional domain and gene structure analysis of foxtail millet *LIM* genes. (**A**) Function domains in foxtail millet LIM proteins. The green pentagon represents the LIM domain, and the gray-black ellipse represents the UIM domain. Members of the PLIM and WLIM subgroups of foxtail millet each contain two LIM domains. Members of the DA1&DAR subgroup contain only one LIM domain and two to three UIM domains. (**B**) Intron–exon structures of *SiLIM* genes. The intron-exon structures were obtained by comparing the coding and genomic sequences using the GSDS website. Yellow and blue boxes represent coding regions and untranslated upstream/downstream regions, respectively. Lines indicate introns. Red represents the *SiWLIM2b* gene.

To further analyze the functional domains of *SiLIM* genes, we used the SMART website to obtain the conserved domains in the foxtail millet LIM proteins. All LIM proteins in the CRP subfamily contain two LIM domains, and members of the DA1&DAR subfamily contain a conserved LIM domain and two to three conserved UIM domains (Figure 2A).

Exon-intron structural divergence within families plays a pivotal role in the evolution of multi-gene families [41]. We used the Gene Structure Display Server (GSDS) to construct a sketch of the exon-intron structure of each *SiLIM* gene. The gene structures are consistent with the classification of the proteins based on the phylogenetic tree. We found that the genes in each subfamily generally had similar exon-intron structures. The six *LIM* genes belonging to the CPR-like subfamily contain four introns, and two of the *LIM* genes in the DA1&DAR subfamily contain 10 introns. The other two of the *LIM* genes in the DA1&DAR subfamily genes contain 11 introns (Figure 2B). The number of exons in the 10 *LIM* genes ranged from 5 to 12 (Figure 2B), and we found examples of exon loss or acquisition during the evolution of the *LIM* genes in foxtail millet. The gain or loss of exons indicates that the function of these genes may change.

### 2.3. Promoter Analysis and Tissue-Specific Expression of the LIM Genes in Foxtail Millet

**Figure 3:**
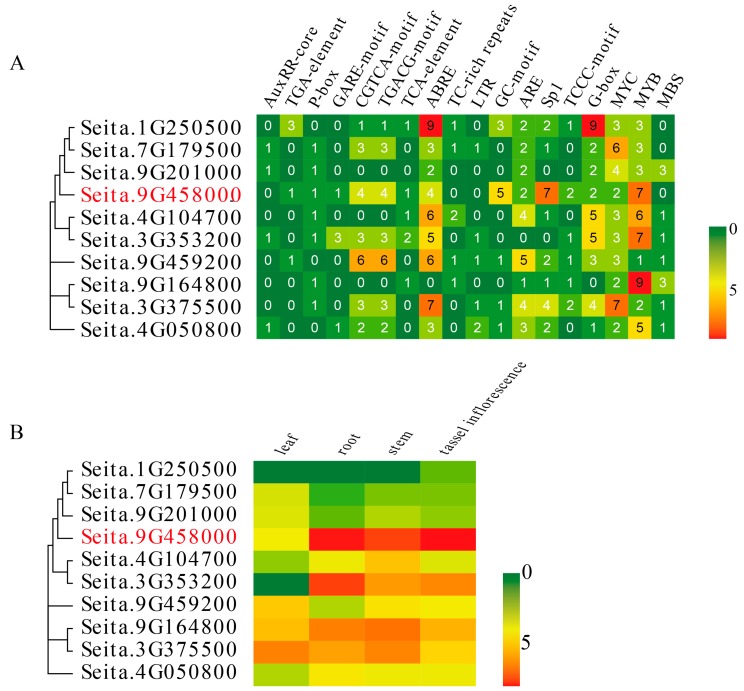
Promoter analysis of foxtail millet *LIM* genes and Tissue-specific expression profiles. (**A**) Promoter analysis of foxtail millet *LIM* genes. The *cis*-acting elements in the promoter regions were obtained from the PlantCARE database. Hormone response elements: AuxRR-core, TGA-element, P-box, GARE-motif, CGTCA-motif, TGACG-motif, TCA-element, ABRE; abiotic stress elements: TC-rich repeats, LTR, GC-motif, ARE, Sp1, TCCC-motif, G-box, MYC, MYB, MBS. Different colors represent the number of *cis*-acting elements within the promoter, with red indicating a higher number and green indicating a lower number. (**B**) Tissue-specific expression data were derived from published Illumina RNA-seq data. Data for roots, stems, leaves and tassel inflorescences were downloaded from the European Nucleotide Archive database [47,48]. The fragments per kilobase of exon model per million mapped reads (FPKM) values were log_2_ transformed, and a heat map was drawn using Evolview (http://www.evolgenius.info/evolview, accessed date: 7 September 2018). Different colors represent the level of expression, where red indicates a high level of gene expression, and green represents a low level of gene expression. The *SiWLIM2b* gene is marked in red.

*Cis*-regulatory elements play an important role in regulating tissue-specific gene expression [42], and also participate in drought response, pathogen defense and cell wall metabolism [43]. Analysis of the foxtail millet *LIM* gene promoters revealed that there are many *cis*-acting elements associated with the response to hormones and abiotic stresses (Figure 3A) and that all of *SiLIM* gene promoters contain MYB and MYC elements, which are related to drought and ABA response (Figure 3A) [44,45,46]. Most *SiLIM* gene promoters have a light-responsive element (Sp1, TCCC-motif, or G-box) and the abscisic acid response element (ABRE, MBS) (Figure 3A). In addition, some foxtail millet *LIM* gene promoters contain hormone response elements, including gibberell in response elements (P-box, GARE-motif), the salicylic acid response element (TCA-element), methyl jasmonate response elements (CGTCA-motif, TGACG-motif) and the low-temperature responsive element (LTR) (Figure 3A). There are differences of *cis*-elements in *LIM* genes from different subgroups. Among the *LIM* genes of CRP-like subgroup of foxtail millet, 83.3% of these genes contain auxin response elements, 83.3% of these genes contain GA response elements and 66.6% of these genes contain SA response elements. Among the *LIM* genes of DA1&DAR subgroup of foxtail millet, 50% of these genes contain auxin response elements, 75% of these genes contain GA response elements and 25% of these genes contain SA response elements, which were significantly lower than the CRP-like subgroup. In the CRP subgroup of *LIM* genes, 33.3% of these genes contain LTR elements, 66.6% of these genes contain JA response elements, while in the DA1-DAR subgroup of *LIM* genes, 75% of these genes contain LTR elements and 75% of these genes contain JA response elements, which is significantly higher than that in the CRP subgroup. More details about the *cis*-elements in *SiLIM* family gene promoters are shown in Supplementary Table S2.

We analyzed the expression profiles of the *LIM* genes in foxtail millet roots, stems, leaves and tassel inflorescences using the RNA-seq data in the European Nucleotide Archive database [47,48] (Figure 3B). This analysis showed that *Seita.1G250500* was specifically expressed in tassel inflorescences and that *Seita.3G353200* was mainly expressed in the root and not expressed in the leaf. The remaining genes were expressed in all four tissues. *Seita.3G375500*, *Seita.7G179500*, *Seita.9G201000* and *Seita.9G459200* were most highly expressed in leaves. The *Seita.9G458000* (*SiWLIM2b*) gene had the highest and lowest expression levels in roots and leaves, respectively. *Seita.4G104700* and *Seita.9G164800* were most highly expressed in stems and *Seita.4G050800* was most highly expressed in roots (Figure 3B). This expression profiling would facilitate combinatorial usage of *SiLIM*s in plant cell processes of different plant tissues, whereas *SiLIMs* expressed in all four tissues may participate in a broad set of plant cell processes. The *seita.9G458000* was more highly expressed in roots and tassels than other nine *LIM* genes, which indicated that *seita.9G458000* plays a more important role in roots and tassel inflorescences.

### 2.4. Analysis of SiWLIM2b Expression under Various Treatments

**Figure 4:**
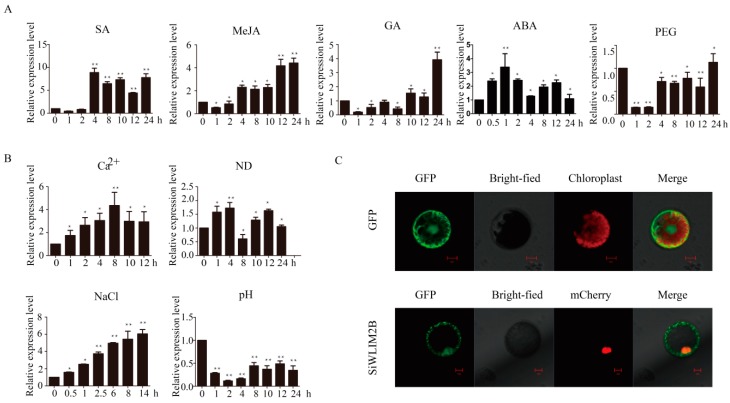
Molecular characteristics of the *SiWLIM2b* gene and Subcellular localization of the SiWLIM2b protein. (**A**) *SiWLIM2b* expression in seedlings treated with ABA, SA, GA, MeJA or PEG. (**B**) *SiWLIM2b* expression in seedlings in the presence of Ca^2+^, nitrogen-deficient conditions, NaCl or high pH. (**C**) Subcellular localization analysis of the SiWLIM2b protein in tobacco protoplasts. The SiWLIM2b-GFP fusion protein is mainly distributedin the cytoplasm and nucleus. Vertical bars in (**B**,**C**) indicate ±SE of three replicates. ** indicates significant differences in comparison with the control lines at *p* < 0.01. * indicates significant differences in comparison with the control lines at *p* < 0.05. (C) Scale bars = 10 μm.

By comparing and analyzing the *cis*-elements in the promoter region of *LIM* genes in foxtail millet, we found that the promoter region of *seita.9G458000* contained the most *cis*-elements related to abiotic stress (27), while the promoter region of *seita.9G201000* contained the least *cis*-elements related to abiotic stress (14). Therefore, we selected the WLIM2 subfamily gene *SiWLIM2b* (Figure 1A) for further study. In order to explore the possible role of *SiWLIM2b* in foxtail millet, we used qRT-PCR to analyze the expression of the *SiWLIM2b* gene in response to different abiotic stresses and exogenous hormones. We found that *SiWLIM2b* was up-regulated in response tosalicylic acid (SA), methyl jasmonate (MeJA), gibberellic acid (GA), abscisic acid (ABA), high Ca^2+^ (120 ppm), and high salt (150 mM NaCl) (Figure 4A,B). *SiWLIM2b* was significantly down-regulated after 2 h of simulated drought (15% PEG) or high pH (8.0) (Figure 4A,B). Under nitrogen deficiency (ND), *SiWLIM2b* was slightly up-regulated (Figure 4B).

### 2.5. Subcellular Location of the SiWLIM2b Protein

In order to identify the subcellular localization of the SiWLIM2b protein, the full-length coding sequence (CDS) of the *SiWLIM2b* gene was cloned into an expression vector containing the GFP tag (16318hGFP) with expression of the SiWLIM2b-GFP fusion protein driven by the CaMV35S promoter. The plasmid SiWLIM2b-GFP was co-transformed into tobacco protoplasts with the nucleus marker AT2G03340-mCherry [49,50]. The SiWLIM2b-GFP fusion protein was mainly distributed in both the cytoplasm and nucleus, and GFP expressed from an empty 35S::GFP expression vector, which served as a control, was mainly distributed in the plasma membrane, cytoplasm and nucleus (Figure 4C). We speculated that the SiWLIM2b protein may act as a transcription factor and may shuttle between the cytoplasm and nucleus.

### 2.6. Overexpression of SiWLIM2b Enhances Drought Resistancein Transgenic Rice

**Figure 5:**
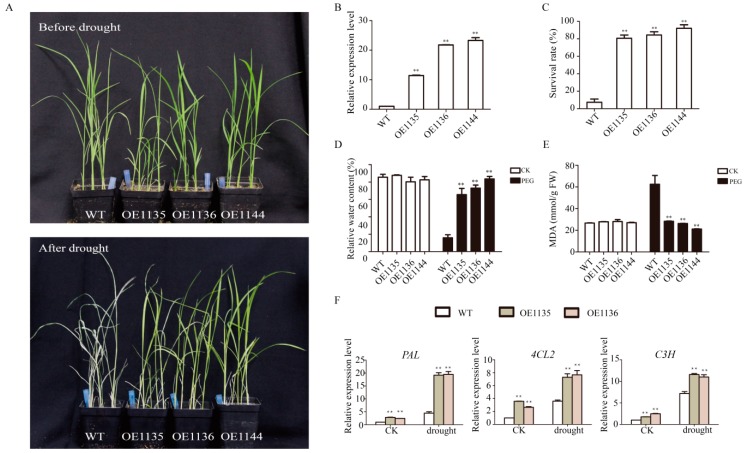
Phenotypic analysis of transgenic and control rice seedlings under drought stress. (**A**) Growth of transgenic and control rice seedlings under normal conditions and after drought treatment for 12 days. (**B**) The expression level of the *SiWLIM2b* gene in transgenic rice lines was determined by qRT-PCR. (**C**) The survival rates of control rice and transgenic rice lines after drought treatment. (**D**) The relative water contents of the control and transgenic rice lines under normal growth conditions and after drought treatment. (**E**) The MDA contents of the control and transgenic rice lines under normal growth conditions and after drought treatment. (**F**) The expression of genes related to the phenylpropane secondary metabolic pathway normal growth (CK) and after drought treatment. Vertical bars in (**B**–**F**) indicate ±SE of three replicates. ** indicates significant differences in comparison with the control lines at *p* < 0.01. * indicates significant differences in comparison with the control lines at *p* < 0.05.

Because the *SiWLIM2b* gene is regulated by drought stress, we wanted to further analyze the functions of *SiWLIM2b* in drought resistance. Phenotypic analysis was performed using homozygous T3 seeds of transgenic rice lines expressing *SiWLIM2b*. We analyzed the tolerance of *SiWLIM2b* transgenic rice under drought stress in a greenhouse and found that three independent transgenic rice lines grew better than wild type (WT) under drought stress (Figure 5A). Three transgenic lines were analyzed. At least 25 seedlings from each line were measured. qRT-PCR showed that the *SiWLIM2b* gene was more highly expressed in the three transgenic rice lines than in WT (*p* < 0.01) (Figure 5B). After drought treatment, WT plants withered and died earlier than the transgenic rice plants, and the survival rate of the transgenic rice lines was higher than that of WT (*p* < 0.01) (Figure 5C). Relative water content analysis showed there was no significant difference between the transgenic lines and WT under normal conditions, whereas under drought conditions, the three transgenic rice lines had significantly higher relative water contents than WT (*p* < 0.01) (Figure 5D). We measured the malondialdehyde (MDA) content of transgenic rice and WT, and found that there was no significant difference in the MDA content under normal conditions, whereas under drought treatment, the MDA content of the three transgenic rice lines was significantly lower than that of WT (*p* < 0.01) (Figure 5E). The lower MDA content indicates that the transgenic lines had a lower degree of membrane damage and higher drought resistance than the WT.

*LIM* genes have been shown to regulate the phenylpropanoid secondary metabolic pathway [5,6,21,29]. Therefore, we analyzed the expression of three genes encoding key rate-limiting enzymes in the phenylpropane pathway: *PAL* (phenylalanine ammonia-lyase), *4CL2* (4-coumarate coenzyme A (CoA) ligase 2) and *C3H* (coumarate 3-hydroxylase) [51]. The qRT-PCR data showed that these phenylpropane synthesis-related genes were more highly expressed in the transgenic rice lines than in WT under both normal growth conditions and under drought treatment (Figure 5F). In addition, the expression levels of the three genes were significantly higher under drought than under normal conditions.

## 3. Discussion

Plants encounter various biotic and abiotic stresses in the stage of growth and development, as one of the members of plant protein family, LIM proteins are heavily studied. Previous research showed that plant LIM proteins are mainly involved in actin cytoskeleton remodeling and the transcriptional regulation of gene expression. Han et al. found that cotton WLIM1a played a role in developing cotton fibers [21]. Recent studies have showed that *LIM* gene expression in plants was induced by various biotic and abiotic stresses. Although the LIM proteins family has been studied extensively in many crops, knowledge about foxtail millet stress tolerance was limited. Here, a genome-wide analysis of the *LIM* family was performed in foxtail millet and 10 *LIM* genes were identified. Among the 10 *LIM* genes, *SiWLIM2b* was screened to study its function in stress responses and this study will provide some information towards further study of the *LIM* gene in other species.

An accurate evolution history is the first step to understanding the evolution relationship of genes, and may provide some useful information to study the pathway and function they are involved in or regulated by [52]. To understand the relationship of foxtail millet LIM proteins with other LIM proteins in different species, a phylogenetic tree of the LIM domain proteins from rice, maize, *Arabidopsis* and foxtail millet was constructed. The result showed that foxtail millet LIM proteins shared genes more closely related with rice and maize LIM proteins than with *Arabidopsis* LIM proteins, which indicated that the LIM gene family differentiated after the divergence of monocotyledons and dicotyledons. These results are consistent with the present understanding of plant evolutionary history [53]. Zhang, G. Y. et al. revealed five major duplications between chromosomes 2 and 9, 4 and 1, 7 and 1, 6 and 2, and 5 and 3 [38]. In this study, chromosomal distributionanalysis of the *LIM* gene family of foxtail millet showed that the *LIM* genesof foxtail millet was only distributed on chromosomes 1, 3, 4, 7 and 9. This may indicate that the formation of *LIM* genes family of foxtail millet occurred after the chromosome duplication events.

The function of a gene is related to its structure, for example, introns can increase transcription levels by affecting the transcription rate, nuclear output and transcript stability [54]. Both the functional domain analysis and exon-intron structure analysis showed that *LIM* genes were conserved in the same subgroup, which indicated that *LIM* genes in the same subgroup have some functional similarities. Both of the functional domains and exon-intron structures of DA1&DAR subfamily genes is more complex than that of CRP subfamily genes, which may suggest that substantial differentiations might have occurred during the evolution of this gene family. The DA1&DAR protein has some roles that are different to CRP-like protein. For example, the DA1 family also plays a role in regulating the degradation of abnormal proteins [55,56] and in the cell cycle [57]. DA1 was also found to regulate the size of seeds and organs in *Arabidopsis* [58,59].

*Cis*-regulatory elements in the promoter regions of genes play significant roles in plant stress responses [60]. Hence, we analyzed the *cis*-acting elements in promoters of the *SiLIM* genes and found elements related to GA, auxin, MeJA, and SA response. These elements play an important role in many physiological processes, such as plant development, senescence and maturation, secondary metabolism and response to various environmental stresses [37,61,62]. For example, the P-box and GARE-motifsare associated with the GA signal transduction pathway, and the CGTCA-motif and TGACG-motif are associated with the response to MeJA [37,61,62]. In addition, we found the ABRE in the promoters of *SiLIM* genes, and confirmed that the bZIP transcription factors AREB1, AREB2, and ABF3 interact with the ABRE motifs in the DREB2A promoter to co-regulate plant responses to osmotic stress [63]. All promoters contained more than one element related to drought and ABA response, such as MYC and MYB elements [44]. Based on this evidence, we speculate that the *SiLIM* genes are probably involved in abiotic stress responses.

The *LIM* genes have been found to be induced by various stresses and hormones in other plants. The expression levels of *PbLIMs* were significantly induced by SA, ABA and MeJA in pear [30]. The *LIM* genes of tomato were induced by ABA, cold, drought, NaCl and heat treatment [22]. The *LIM* genes of Brassica rapa were induced by cold, ABA and pH (pH5, pH7 and pH9) treatments [64]. Similar results were found in this study. QPCR analysis showed that the *SiWLIM2b* gene was induced by various stresses (PEG, Ca^2+^, ND, NaCl, pH) and exogenous hormones (ABA, SA, MeJA, GA). These studies may illustrate that the functions of *LIM* genes in different species appear to be similar. We chose the *SiWLIM2b* gene for further study and found that *SiWLIM2b* was induced by drought. In order to further study the role of *SiWLIM2b* in drought resistance, we studied the phenotype of transgenic rice heterologously by expressing the *SiWLIM2b* gene under drought conditions. We found that the *SiWLIM2b* gene can improve drought resistance when expressed in transgenic rice (Figure 5). MDA can inhibit the activity of cell protective enzymes and reduce the antioxidant content [65,66]. When the enzymes and membrane systems of plant tissues are destroyed, the MDA content is greatly increased. Therefore, MDA reflects the antioxidant capacity of plant tissues, and can also serve as a measure of plant resistance to external stresses [67]. Determination of the MDA content of transgenic lines and WT under drought stress showed that under drought stress transgenic lines had much less membrane damage and higher drought resistance than the kitaake (WT) [65,67]. These results indicate that the *SiWLIM2b* gene is involved in drought response and could be used as a new candidate gene in molecular breeding for crop stress resistance.

A recent study reported that the function of LIM proteins is closely related to its subcellular localization and LIM proteins with nuclear localized or co-localized in the cytoplasm and nucleus act in regulation of gene expression [6,14,21]. In this study, we found that the SiWLIM2b protein mainly accumulated in both the cytoplasm and nucleus, suggesting that it may function in the regulation of gene expression. In our study, the expression of three genes encoding key rate-limiting enzymes in the phenylpropane pathway was up-regulated in the transgenic rice lines. This suggests that *SiWLIM2b* gene may be involved in the phenylpropane pathway. It was confirmed that the synthesis substances of the phenylpropanol pathway greatly enhance the tolerance to drought in plants [24]. Wei et al. confirmed that the *TaMyb1D* gene in transgenic tobacco regulated the expression of related genes in the phenylpropanol metabolism pathway, and over-expression of *TaMyb1D* enhanced drought resistance of transgenic tobacco [68]. Similar results were found in our study, three phenylpropane synthesis-related genes were up-regulated expression in transgenic rice seedlings after drought treatment. This suggests that over-expression of the *SiWLIM2b* gene may enhance drought resistance of transgenic rice by participating in the secondary metabolic pathway of phenylpropane. However, we found that *SiWLIM2b* did not have transcriptional activation activity (Supplemental Figure S1). We speculate that *SiWLIM2b* may also regulate the expression of genes by interacting with other transcription factors [28]. Further experiments are required to identify the relationship between the *SiWLIM2b* gene and the phenylpropanoid pathway and drought resistance.

## 4. Materials and Methods 

### 4.1. Plant Materials

Foxtail millet seeds were grown in nutrient soil (1:1 mix of nutrient soil and vermiculite) at 28 °C for three weeks, and then subjected tovarious abiotic stresses, namely drought (15% PEG), high salinity (150 mM NaCl), high pH (8.0) and high Ca^2+^ (120 ppm), or treatment with exogenous hormones, namely ABA (20 μM), SA (20 μM), GA (20 μM) and MeJA (20 μM). Seedlings were sampled at different times after being transferred to a solution containing each substance. In order to subject foxtail millet seedlings to low nitrogen conditions (ND treatment), foxtail millet seeds were germinated at 28°C for 2 days, then transferred into Hoagland nutrient solution and grown to the four-leaf stage. Seedlings were then transferred into nitrogen-free nutrient solution and sampled after 0, 1, 4, 8, 10, 12 and 24 h. All samples were frozen in liquid nitrogen and stored at −80 °C.

### 4.2. Identification and Phylogenetic Analysis of LIMFamily Genes

The Hidden Markov Model (HMM) of the LIM domain (PF00412) was obtained from Pfam 31.0 (https://en.wikipedia.org/wiki/Pfam, accessed date: 11 August 2018). The putative *LIM* genes were identified in the foxtail millet genomic database (Phytozome v12.1, https://phytozome.jgi.doe.gov/pz/portal.html#, accessed date: 11 August 2018) using ‘LIM’ and ‘PF00412’ as key wordswith a threshold E values ≤ 1.0. Each putative *SiLIM* gene sequence was checked using SMART (http://smart.embl-heidelberg.de/, accessed date: 11 August 2018) to confirm the presence of the LIM domain. The protein sequences of the *Arabidopsis LIM* genes were acquired from TAIR (https://www.arabidopsis.org/, accessed date: 11 August 2018) and those of the rice and maize *LIM* genes were acquired from the Phytozome database (https://phytozome.jgi.doe.gov/pz/portal.html#, accessed date: 11 August 2018). In order to investigate the evolutionary relationships among the LIM domain proteins in different plant species, aphylogenetic tree was constructed [69]. All *Arabidopsis*, rice, maize and foxtail millet LIM protein sequences were imported using MEGA7.0 [70], and multiple sequence alignments were performed using ClustalW with the default multiple alignment parameters. The phylogenetic tree was constructed using the maximum likelihood method. Information of LIM indifferent species (*Arabidopsis*, rice, maize) is listed in Supplemental Table S3). The percentage of similarity compared with the sequences of LIM proteins in foxtail millet and the LIM proteins of maize, rice and arabidopsis thaliana on the same evolutionary branch is listed in Supplemental Table S4.

### 4.3. Chromosome Locations and Gene Structures of LIM Family Genes and Identification of Functional Domains and Cis-Acting Elements

Foxtail millet chromosome lengths were obtained from the file Setaria_italica.JGIv2.0.38.gff3, and the position of each *LIM* gene on the foxtail millet chromosomes was obtained from Phytozome (https://phytozome.jgi.doe.gov/pz/portal.html#, accessed date: 11 August 2018). All *SiLIM* genes were mapped onto the nine foxtail millet chromosomes according their physical position (bp) using MapGene2Chromosome v2 (http://mg2c.iask.in/mg2c_v2.0/, accessed date: 12 August 2018). The exon-intron structures of the *LIM* genes were determined by comparing the CDS with the genomic sequences using the GSDS (http://gsds.cbi.pku.edu.cn/, accessed date: 12 August 2018) [71].

The SMART online tool (http://smart.embl-heidelberg.de/, accessed date: 11 August 2018) was used to analyze the functional domains in the SiLIM proteins, and functional domain sketches were drawn using the ExPAsy-PROSITE website (https://prosite.expasy.org/, accessed date: 13 August 2018). The molecular weights and the oretical isoelectric points of the SiWLIMs were calculated using the ExPASy online tool (https://web.expasy.org/protparam/, accessed date: 13 August 2018).

To obtain the promoter sequences we downloaded approximately 2.0 Kb of sequence upstream of each *SiLIM* coding sequence from the Phytozome database. *Cis*-acting elements were analyzed using the plant *cis*-acting element database PlantCARE (http://bioinformatics.psb.ugent.be/webtools/plantcare/html/, accessed date: 15 August 2018) [72]. Heat map showing the number of *cis*-acting elements in the promoters were made using EVOLVIEW (http://www.evolgenius.info/evolview/#mytrees//, accessed date: 7 September 2018) [73].

### 4.4. Tissue-Specific Expression Profiling Using RNA-Seq Data

Illumina RNA-seq data for four tissues namely roots, stems, leaves and tassel inflorescences were downloaded from the European Nucleotide Archive database [SRX128223 (root); SRX12825 (stem); SRX128224 (leaf); SRX128226 (spica)] [47,48]. Four tissues were obtained from young seedlings of Zhang-gu, which grew in nutrient soil for 40 days in a greenhouse [38]. RNA-seq data were mapped onto the gene sequences of foxtail millet using CLC Genomics Workbench V4.7.1 tooland normalized by FPKM (reads per kilobase per million). A heat map showing tissue-specific expression profiles (log_2_RPKM values) was made using EVOLVIEW. The expression profiling data are shown in Supplementary Table S5.

### 4.5. Total RNA Extraction and qRT-PCR Analysis

Total RNA was extracted from foxtail millet using the Plant Total RNA Kit (Zhuangmeng, Beijing, China) according to the manufacturer’s manual. The extracted RNA was detected by electrophoresis on a 1% agarose gel, and the first strand of cDNA was synthesized using the EasyScript One-Step gDNA Removal and cDNA Synthesis SuperMix kit (TransGen Biotech, Beijing, China). The relative expression level of the *SiWLIM2b* gene under various stresses was analyzed by qRT-PCR. The Primer3 website (http://bioinfo.ut.ee/primer3-0.4.0/, accessed date: 4 November 2017) was used to design primers for qRT-PCR [74,75], and the 2^−ΔΔCt^ method was used to calculate the relative expression level ofthe *SiWLIM2b* gene. The actin gene (Genbank number: AF288226) of foxtail millet was used as an internal control. QRT-PCR was performed for three biological replicates, with three technical replicates per biological replicate. All primer sequences used are shown in Supplementary Table S6.

### 4.6. Subcellular Localization of SiWLIM2b

We constructed a vector expressing a GFP-tagged fusion protein for subcellular localization analysis of the SiWLIM2b protein. A cDNA containing the coding sequence of *SiWLIM2b* was cloned into the 16318h GFP vector and expressed under the control of the CaMV35s promoter. The recombinant plasmids were confirmed by sequencing. The full-length cDNA coding sequences of AT2G03340 which were located in the nucleus [49] were cloned into the mCherry ORF (WRKY25-RFP) under the control of the CaMV 35S promoter [50]. The plasmid SiWLIM2b-GFP and AT2G03340-mcherry were co-transformed into tobacco protoplasts using the PEG-mediated method and observed under a confocal microscope 16h after transformation [76]. The 35S::GFP vector was transformed as the control.

### 4.7. Phenotypic Analysis of Transgenic Rice in a Greenhouse

The *SiWLIM2b* complete open reading frame (ORF) was amplified by PCR usingthe primers F1 (BamH I) and R1 (Sac I). The amplified PCR fragment was cloned into vector pMWB014 (driven by the ubiquitin promoter) digested with BamH I and Sac I. The pMWB014-*SiWLIM2b* was confirmed by sequencing and transformed into the kitaake rice (WT) cultivar by Agrobacterium-mediated transformation. The transformed callus was cultured on medium with 1.0 mg/L glufosinate. Six transgenic rice lines were obtained by using primers F2 (designed according to the CDS of *SiWLIM2b* gene) and R2 (designed according to NOS terminator sequence) for PCR verification. The resulting transgenic rice was cultured to the T3 generation. We selected three transgenic lines in the T3 generation (OE1135, OE1136, and OE1144) for subsequent functional analysis. To determine whether transgenic rice seedlings could resist drought stress, we conducted a pot experiment in the greenhouse. Rice seeds were soaked with 2.5% sodium hypochlorite (NaClO) for 30 min, then rinsed with tap water 5–6 times. Sterilized seeds were germinated in a 28-degree incubator, and the water was changed every 12 h. After germination, the seeds wereplanted in nutrient soil and grown to the three-leaf stage under a 16-h/8-h photoperiod at 30 °C. Rice seedlings were then subjected to drought treatment for 12 days and survival was scored by dividing the number of plants alive after drought treatment for 12 days by the total number of plants surveyed, using one green leaf as the standard for survival. Relative water content [77,78] and MDA content [66] were determined in transgenic and control rice after 5 days of drought treatment. All experiments were set up with three independent biological replicates.

### 4.8. Differential Expression of Phenylpropane Secondary Metabolic Pathway Genes in Transgenic and Control Rice Plants

Expression of phenylpropane secondary metabolic pathway-related genes in control and transgenic rice was analyzed by qRT-PCR. Rice were grown to the four-leaf stage in Hoagland nutrient solution, then treated with 6% PEG for three days. RNA was extracted and reverse transcribed into cDNA, which was used as template for qRT-PCR. Primers used in qRT-PCR are listed in Supplemental Table S4.

## 5. Conclusions

A total of 10 *LIM* protein-coding genes were identified in foxtail millet via a genome-wide analysis. Analysis of evolutionary relationships indicated that *SiLIM* genes could be divided into two large subgroups. The chromosome positions, gene structures, functional domains and promoters of the *SiLIM* genes were also analyzed. The *SiWLIM2b* gene was found to respond to various abiotic stresses and hormone treatments, and overexpression of the *SiWLIM2b* gene in rice improved drought tolerance.

## Supplementary Materials

Supplementary Materials can be found at https://www.mdpi.com/1422-0067/20/6/1303/s1.

## Abbreviations

*PAL*	Phe ammonia lyase
ROS	Reactive oxygen species
MDA	malondialdehyde
*4CL2*	4-coumarate coenzyme A (CoA) ligase 2
*C3H*	Coumarate-3-hydroxylase
SA	salicylic acid
MeJA	methyl jasmonate
GA	gibberellic acid
ABRE	ABA-responsive element
qRT-PCR	quantitative real-time PCR
GFP	green fluorescent protein
ND	nitrogen deficiency
ABA	abscisic acid

## References

[1] Eliasson A., Gass N., Mundel C., Baltz R., Krauter R., Evrard J.L., Steinmetz A. (2000). Molecular and expression analysis of a lim protein gene family from flowering plants. Mol. Gen. Genet..

[2] Kadrmas J.L., Beckerle M.C. (2004). The lim domain: From the cytoskeleton to the nucleus. Nat. Rev. Mol. Cell Biol..

[3] Srivastava V., Verma P.K. (2017). The plant lim proteins: Unlocking the hidden attractions. Planta.

[4] Wang H.J., Wan A.R., Jauh G.Y. (2008). An actin-binding protein, lllim1, mediates calcium and hydrogen regulation of actin dynamics in pollen tubes. Plant Physiol..

[5] Kawaoka A., Ebinuma H. (2001). Transcriptional control of lignin biosynthesis by tobacco lim protein. Phytochemistry.

[6] Kawaoka A., Kaothien P., Yoshida K., Endo S., Yamada K., Ebinuma H. (2000). Functional analysis of tobacco lim protein ntlim1 involved in lignin biosynthesis. Plant J. Cell Mol. Biol..

[7] Labalette C., Nouet Y., Levillayer F., Colnot S., Chen J., Claude V., Huerre M., Perret C., Buendia M.A., Wei Y. (2010). Deficiency of the lim-only protein fhl2 reduces intestinal tumorigenesis in apc mutant mice. PLoS ONE.

[8] Way J.C., Chalfie M. (1988). Mec-3, a homeobox-containing gene that specifies differentiation of the touch receptor neurons in c. Elegans. Cell.

[9] Freyd G., Kim S.K., Horvitz H.R. (1990). Novel cysteine-rich motif and homeodomain in the product of the caenorhabditis elegans cell lineage gene lin-11. Nature.

[10] Karlsson O., Thor S., Norberg T., Ohlsson H., Edlund T. (1990). Insulin gene enhancer binding protein isl-1 is a member of a novel class of proteins containing both a homeo- and a cys-his domain. Nature.

[11] Dawid I.B., Toyama R., Taira M. (1995). Lim domain proteins. C. R. l’Acad. Sci. Ser. III Sci..

[12] Perez-Alvarado G.C., Miles C., Michelsen J.W., Louis H.A., Winge D.R., Beckerle M.C., Summers M.F. (1994). Structure of the carboxy-terminal lim domain from the cysteine rich protein crp. Nat. Struct. Biol..

[13] Baltz R., Evrard J.L., Bourdon V., Steinmetz A. (1996). The pollen-specific lim protein plim-1 from sunflower binds nucleic acids in vitro. Sex. Plant Reprod..

[14] Moes D., Gatti S., Hoffmann C., Dieterle M., Moreau F., Neumann K., Schumacher M., Diederich M., Grill E., Shen W.H. (2013). A lim domain protein from tobacco involved in actin-bundling and histone gene transcription. Mol. Plant.

[15] Thomas C., Hoffmann C., Dieterle M., Van Troys M., Ampe C., Steinmetza A. (2006). Tobacco wlim1 is a novel f-actin binding protein involved in actin cytoskeleton remodeling. Plant Cell.

[16] Papuga J., Thomas C., Dieterle M., Moreau F., Steinmetz A. (2009). Arabidopsis lim domain proteins involved in actin bundling exhibit different modes of regulation. FEBS J..

[17] Papuga J., Hoffmann C., Dieterle M., Moes D., Moreau F., Tholl S., Steinmetz A., Thomas C. (2010). Arabidopsis lim proteins: A family of actin bundlers with distinct expression patterns and modes of regulation. Plant Cell.

[18] Arnaud D., Dejardin A., Leple J.C., Lesage-Descauses M.C., Pilate G. (2007). Genome-wide analysis of lim gene family in populus trichocarpa, arabidopsis thaliana, and oryza sativa. DNA Res. Int. J. Rapid Publ. Rep. Genes Genomes.

[19] Li Y., Jiang J., Li L., Wang X.L., Wang N.N., Li D.D., Li X.B. (2013). A cotton lim domain-containing protein (ghwlim5) is involved in bundling actin filaments. Plant Physiol. Biochem..

[20] Li L., Li Y., Wang N.N., Li Y., Lu R., Li X.B. (2015). Cotton lim domain-containing protein ghplim1 is specifically expressed in anthers and participates in modulating f-actin. Plant Biol..

[21] Han L.B., Li Y.B., Wang H.Y., Wu X.M., Li C.L., Luo M., Wu S.J., Kong Z.S., Pei Y., Jiao G.L. (2013). The dual functions of wlim1a in cell elongation and secondary wall formation in developing cotton fibers. Plant Cell.

[22] Khatun K., Robin A.H.K., Park J.I., Ahmed N.U., Kim C.K., Lim K.B., Kim M.B., Lee D.J., Nou I.S., Chung M.Y. (2016). Genome-wide identification, characterization and expression profiling of lim family genes in solanum lycopersicum L.. Plant Physiol. Biochem..

[23] Zhao M., He L., Gu Y., Wang Y., Chen Q., He C. (2014). Genome-wide analyses of a plant-specific lim-domain gene family implicate its evolutionary role in plant diversification. Genome Biol. Evol..

[24] Vogt T. (2010). Phenylpropanoid biosynthesis. Mol. Plant..

[25] Fraser C.M., Chapple C. (2011). The phenylpropanoid pathway in arabidopsis. Arabidopsis Book.

[26] Zhang X., Liu C.J. (2015). Multifaceted regulations of gateway enzyme phenylalanine ammonia-lyase in the biosynthesis of phenylpropanoids. Mol. Plant.

[27] Sala S., Ampe C. (2018). An emerging link between lim domain proteins and nuclear receptors. Cell. Mol. Life Sci..

[28] Zheng Q., Zhao Y. (2007). The diverse biofunctions of lim domain proteins: Determined by subcellular localization and protein-protein interaction. Biol. Cell.

[29] Kaothien P., Kawaoka A., Ebinuma H., Yoshida K., Shinmyo A. (2002). Ntlim1, a pal-box binding factor, controls promoter activity of the horseradish wound-inducible peroxidase gene. Plant Mol. Biol..

[30] Cheng X., Li G., Muhammad A., Zhang J., Jiang T., Jin Q., Zhao H., Cai Y., Lin Y. (2018). Molecular identification, phylogenomic characterization and expression patterns analysis of the lim (lin-11, isl1 and mec-3 domains) gene family in pear (pyrus bretschneideri) reveal its potential role in lignin metabolism. Gene.

[31] Wasteneys G.O., Yang Z. (2004). The cytoskeleton becomes multidisciplinary. Plant Physiol..

[32] Wang C., Zhang L.J., Huang R.D. (2011). Cytoskeleton and plant salt stress tolerance. Plant Signal. Behav..

[33] Dixon R.A., Paiva N.L. (1995). Stress-induced phenylpropanoid metabolism. Plant Cell.

[34] Yamaguchi M., Valliyodan B., Zhang J., Lenoble M.E., Yu O., Rogers E.E., Nguyen H.T., Sharp R.E. (2010). Regulation of growth response to water stress in the soybean primary root. I. Proteomic analysis reveals region-specific regulation of phenylpropanoid metabolism and control of free iron in the elongation zone. Plant Cell Environ..

[35] Fang Y.J., Xiong L.Z. (2015). General mechanisms of drought response and their application in drought resistance improvement in plants. Cell. Mol. Life Sci..

[36] Lata C., Gupta S., Prasad M. (2013). Foxtail millet: A model crop for genetic and genomic studies in bioenergy grasses. Crit. Rev. Biotechnol..

[37] Yu T.F., Zhao W.Y., Fu J.D., Liu Y.W., Chen M., Zhou Y.B., Ma Y.Z., Xu Z.S., Xi Y.J. (2018). Genome-wide analysis of cdpk family in foxtail millet and determination of sicdpk24 functions in drought stress. Front. Plant Sci..

[38] Zhang G., Liu X., Quan Z., Cheng S., Xu X., Pan S., Xie M., Zeng P., Yue Z., Wang W. (2012). Genome sequence of foxtail millet (setaria italica) provides insights into grass evolution and biofuel potential. Nat. Biotechnol..

[39] Bennetzen J.L., Schmutz J., Wang H., Percifield R., Hawkins J., Pontaroli A.C., Estep M., Feng L., Vaughn J.N., Grimwood J. (2012). Reference genome sequence of the model plant setaria. Nat. Biotechnol..

[40] Jiangtao C., Yingzhen K., Qian W., Yuhe S., Daping G., Jing L., Guanshan L. (2015). Mapgene2chrom, a tool to draw gene physical map based on perl and svg languages. Yi Chuan (Hereditas).

[41] Zhang Y., Mao L., Wang H., Brocker C., Yin X., Vasiliou V., Fei Z., Wang X. (2012). Genome-wide identification and analysis of grape aldehyde dehydrogenase (aldh) gene superfamily. PLoS ONE.

[42] Wittkopp P.J., Kalay G. (2011). Cis-regulatory elements: Molecular mechanisms and evolutionary processes underlying divergence. Nat. Rev. Genet..

[43] Wong D.C.J., Lopez Gutierrez R., Gambetta G.A., Castellarin S.D. (2017). Genome-wide analysis of cis-regulatory element structure and discovery of motif-driven gene co-expression networks in grapevine. DNA Res. Int. J. Rapid Publ. Rep. Genes Genomes.

[44] Zhu C.F., Schraut D., Hartung W., Schaffner A.R. (2005). Differential responses of maize mip genes to salt stress and aba. J. Exp. Bot..

[45] Faraji S., Rasouli S.H., Kazemitabar S.K. (2018). Genome-wide exploration of c2h2 zinc finger family in durum wheat (triticum turgidum ssp durum): Insights into the roles in biological processes especially stress response. Biometals.

[46] Onishi M., Tachi H., Kojima T., Shiraiwa M., Takahara H. (2006). Molecular cloning and characterization of a novel salt-inducible gene encoding an acidic isoform of pr-5 protein in soybean (glycine max [L.] merr.). Plant Physiol. Biochem..

[47] Cochrane G., Alako B., Amid C., Bower L., Cerdeno-Tarraga A., Cleland I., Gibson R., Goodgame N., Jang M., Kay S. (2013). Facing growth in the european nucleotide archive. Nucleic Acids Res..

[48] Muthamilarasan M., Bonthala V.S., Mishra A.K., Khandelwal R., Khan Y., Roy R., Prasad M. (2014). C2h2 type of zinc finger transcription factors in foxtail millet define response to abiotic stresses. Funct. Integr. Genom..

[49] Miller M.J., Barrett-Wilt G.A., Hua Z., Vierstra R.D. (2010). Proteomic analyses identify a diverse array of nuclear processes affected by small ubiquitin-like modifier conjugation in arabidopsis. Proc. Natl. Acad. Sci. USA.

[50] Du Y.T., Zhao M.J., Wang C.T., Gao Y., Wang Y.X., Liu Y.W., Chen M., Chen J., Zhou Y.B., Xu Z.S. (2018). Identification and characterization of gmmyb118 responses to drought and salt stress. BMC Plant Biol..

[51] Liu J.Y., Osbourn A., Ma P.D. (2015). Myb transcription factors as regulators of phenylpropanoid metabolism in plants. Mol. Plant.

[52] Shen X.X., Salichos L., Rokas A. (2016). A genome-scale investigation of how sequence, function, and tree-based gene properties influence phylogenetic inference. Genome Biol. Evol..

[53] Kellogg E.A. (2001). Evolutionary history of the grasses. Plant Physiol..

[54] Shaul O. (2017). How introns enhance gene expression. Int. J. Biochem. Cell Biol..

[55] Yan N., Doelling J.H., Falbel T.G., Durski A.M., Vierstra R.D. (2000). The ubiquitin-specific protease family from arabidopsis. Atubp1 and 2 are required for the resistance to the amino acid analog canavanine. Plant Physiol..

[56] Raasi S., Wolf D.H. (2007). Ubiquitin receptors and erad: A network of pathways to the proteasome. Semin. Cell Dev. Biol..

[57] King R.W., Deshaies R.J., Peters J.M., Kirschner M.W. (1996). How proteolysis drives the cell cycle. Science.

[58] Li Y., Zheng L., Corke F., Smith C., Bevan M.W. (2008). Control of final seed and organ size by the da1 gene family in arabidopsis thaliana. Genes Dev..

[59] Xia T., Li N., Dumenil J., Li J., Kamenski A., Bevan M.W., Gao F., Li Y. (2013). The ubiquitin receptor da1 interacts with the e3 ubiquitin ligase da2 to regulate seed and organ size in arabidopsis. Plant Cell.

[60] Zhao W., Liu Y.W., Zhou J.M., Zhao S.P., Zhang X.H., Min D.H. (2016). Genome-wide analysis of the lectin receptor-like kinase family in foxtail millet (setaria italica L.). Plant Cell Tissue Organ Culture.

[61] Pieterse C.M.J., van Wees S.C.M., van Pelt J.A., Knoester M., Laan R., Gerrits N., Weisbeek P.J., van Loon L.C. (1998). A novel signaling pathway controlling induced systemic resistance in arabidopsis. Plant Cell.

[62] Wang Y., Liu G.J., Yan X.F., Wei Z.G., Xu Z.R. (2011). Meja-inducible expression of the heterologous jaz2 promoter from arabidopsis in populus trichocarpa protoplasts. J. Plant Dis. Prot..

[63] Kim J.S., Mizoi J., Yoshida T., Fujita Y., Nakajima J., Ohori T., Todaka D., Nakashima K., Hirayama T., Shinozaki K. (2011). An abre promoter sequence is involved in osmotic stress-responsive expression of the dreb2a gene, which encodes a transcription factor regulating drought-inducible genes in arabidopsis. Plant Cell Physiol..

[64] Park J.I., Ahmed N.U., Jung H.J., Arasan S.K., Chung M.Y., Cho Y.G., Watanabe M., Nou I.S. (2014). Identification and characterization of lim gene family in brassica rapa. BMC Genom..

[65] Gawel S., Wardas M., Niedworok E., Wardas P. (2004). [malondialdehyde (mda) as a lipid peroxidation marker]. Wiadomosci Lekarskie.

[66] Tsikas D. (2017). Assessment of lipid peroxidation by measuring malondialdehyde (mda) and relatives in biological samples: Analytical and biological challenges. Anal. Biochem..

[67] Karatas I., Ozturk L., Demir Y., Unlukara A., Kurunc A., Duzdemir O. (2014). Alterations in antioxidant enzyme activities and proline content in pea leaves under long-term drought stress. Toxicol. Ind. Health.

[68] Wei Q., Zhang F., Sun F., Luo Q., Wang R., Hu R., Chen M., Chang J., Yang G., He G. (2017). A wheat myb transcriptional repressor tamyb1d regulates phenylpropanoid metabolism and enhances tolerance to drought and oxidative stresses in transgenic tobacco plants. Plant Sci. Int. J. Exp. Plant Biol..

[69] Mahapatro G., Mishra D., Shaw K., Mishra S., Jena T. (2012). Phylogenetic tree construction for DNA sequences using clustering methods. Procedia Eng..

[70] Kumar S., Nei M., Dudley J., Tamura K. (2008). Mega: A biologist-centric software for evolutionary analysis of DNA and protein sequences. Brief. Bioinform..

[71] Hu B., Jin J.P., Guo A.Y., Zhang H., Luo J.C., Gao G. (2015). Gsds 2.0: An upgraded gene feature visualization server. Bioinformatics.

[72] Lescot M., Dehais P., Thijs G., Marchal K., Moreau Y., Van de Peer Y., Rouze P., Rombauts S. (2002). Plantcare, a database of plant cis-acting regulatory elements and a portal to tools for in silico analysis of promoter sequences. Nucleic Acids Res..

[73] He Z.L., Zhang H.K., Gao S.H., Lercher M.J., Chen W.H., Hu S.N. (2016). Evolview v2: An online visualization and management tool for customized and annotated phylogenetic trees. Nucleic Acids Res..

[74] Koressaar T., Remm M. (2007). Enhancements and modifications of primer design program primer3. Bioinformatics.

[75] Untergasser A., Cutcutache I., Koressaar T., Ye J., Faircloth B.C., Remm M., Rozen S.G. (2012). Primer3—New capabilities and interfaces. Nucleic Acids Res..

[76] Yoo S.D., Cho Y.H., Sheen J. (2007). Arabidopsis mesophyll protoplasts: A versatile cell system for transient gene expression analysis. Nat. Protoc..

[77] Jungklang J., Saengnil K., Uthaibutra J. (2017). Effects of water-deficit stress and paclobutrazol on growth, relative water content, electrolyte leakage, proline content and some antioxidant changes in curcuma alismatifolia gagnep. Cv. Chiang mai pink. Saudi J. Biol. Sci..

[78] Smart R.E. (1974). Rapid estimates of relative water content. Plant Physiol..

