# Supplementary material for: Genome-Wide Analysis of LIM Family Genes in Foxtail Millet (Setaria italica L.) and Characterization of the Role of SiWLIM2b in Drought Tolerance
Source: Int J Mol Sci. 2019 Mar 15;20(6):1303. doi: 10.3390/ijms20061303 (PMC6470693; doi:10.3390/ijms20061303)
Supplement: Supplementary file 1 [file ijms-20-01303-s001.zip › ijms-442293-Supplemental/Supplemental Table S2 .docx]

|  | | **Seita.9G**  **201000.1** | **Seita.9G**  **164800.1** | **Seita.3G**  **375500.1** | **Seita.9G**  **459200.1** | **Seita.4G**  **050800.1** | **Seita.1G**  **250500.1** | **Seita.4G**  **104700.1** | **Seita.3G**  **353200.1** | **Seita.7G**  **179500.1** | **Seita.9G**  **458000.1** |
| --- | --- | --- | --- | --- | --- | --- | --- | --- | --- | --- | --- |
| **auxin-responsive** | **AuxRR-core** | **1** |  |  |  | **1** |  |  | **1** | **1** |  |
|  | **TGA-element** |  |  |  | **1** |  | **3** |  |  |  | **1** |
| **gibberellin-responsive** | **P-box** | **1** | **1** | **1** |  |  |  | **1** | **1** | **1** | **1** |
|  | **GARE-motif** |  |  |  |  | **1** |  |  | **3** |  | **1** |
| **MeJA-responsiveness** | **CGTCA-motif** |  |  | **3** | **6** | **2** | **1** |  | **3** | **3** | **4** |
|  | **TGACG-motif** |  |  | **3** | **6** | **2** | **1** |  | **3** | **3** | **4** |
| **TCA-element**  **Salicylic acid responsiveness** | |  | **1** |  |  |  | **1** | **1** | **2** |  | **1** |
| **ABRE**  **abscisic acid responsiveness** | | **2** |  | **7** | **6** | **3** | **9** | **6** | **5** | **3** | **4** |

**Supplementary Table S2. Distribution of *cis*-acting elements related hormone responsive in foxtail millet**

RAV gene promoters.

|  | | **Seita.9G**  **201000.1** | **Seita.9G**  **164800.1** | **Seita.3G**  **375500.1** | **Seita.9G**  **459200.1** | **Seita.4G**  **050800.1** | **Seita.1G**  **250500.1** | **Seita.4G**  **104700.1** | **Seita.3G**  **353200.1** | **Seita.7G**  **179500.1** | **Seita.9G**  **458000.1** |
| --- | --- | --- | --- | --- | --- | --- | --- | --- | --- | --- | --- |
| **TC-rich repeats**  **defense and stress responsiveness** | |  | **1** |  | **1** |  | **1** | **2** |  | **1** |  |
| **LTR**  **low-temperature responsiveness** | |  |  | **1** | **1** | **2** |  |  | **1** | **1** |  |
| **anaerobic induction** | **GC-motif** |  |  | **1** | **1** | **1** | **3** |  |  |  | **5** |
|  | **ARE** | **2** | **1** | **4** | **5** | **3** | **2** | **4** |  | **2** | **2** |
| **Light response** | **Sp1** |  | **1** | **4** | **2** | **2** | **2** | **1** |  | **1** | **7** |
|  | **TCCC-motif** |  | **1** | **2** | **1** |  | **1** |  | **1** |  | **2** |
|  | **G-box** | **2** |  | **4** | **3** | **1** | **9** | **5** | **5** | **2** | **2** |
| **MYC**  **drought and ABA responses** | | **4** | **2** | **7** | **3** | **2** | **3** | **3** | **3** | **6** | **2** |
| **MYB**  **drought, ABA, and GA stress responses** | | **3** | **9** | **2** | **1** | **5** | **3** | **6** | **7** | **3** | **7** |
| **MBS**  **drought-inducibility** | | **3** | **3** | **1** | **1** | **1** |  | **1** | **1** |  |  |

**Supplementary Table S2. Distribution of *cis*-acting elements related abiotic stress in foxtail millet**

RAV gene promoters.
