# Supplementary material for: Genome-Wide Analysis of LIM Family Genes in Foxtail Millet (Setaria italica L.) and Characterization of the Role of SiWLIM2b in Drought Tolerance
Source: Int J Mol Sci. 2019 Mar 15;20(6):1303. doi: 10.3390/ijms20061303 (PMC6470693; doi:10.3390/ijms20061303)
Supplement: Supplementary file 1 [file ijms-20-01303-s001.zip › ijms-442293-Supplemental/Supplemental Table S3.docx]

**Supplemental Table S3. Information of different species for sequence alignment and phylogenetic tree**

| Gene names | Locus name |
| --- | --- |
| OsLIM | LOC_Os06g13030.1 |
| OsWLIM1 | LOC_Os12g32620.1 |
| OsWLIM2 | LOC_Os03g15940.1 |
| OsPLIM2a | LOC_Os02g42820.1 |
| OsPLIM2b | LOC_Os04g45010.1 |
| OsPLIM2c | LOC_Os10g35930.1 |
| OsDAR1 | LOC_Os06g08400.1 |
| OsDAR2 | LOC_Os03g16090.1 |
| OsDAR4 | LOC_Os12g40490.1 |
| OsDAR3 | LOC_Os03g42820.1 |
| ZmLIM4 | GRMZM2G170034_T01 |
| ZmPLIM2a | GRMZM2G134752_T01 |
| ZmPLIM2b | GRMZM2G024887_T01 |
| ZmWLIM1 | GRMZM2G004959_T01 |
| ZmWLIM2B | GRMZM2G128206_T03 |
| ZmDAR1 | GRMZM2G342105_T02 |
| ZmDAR2a | GRMZM2G160198_T01 |
| ZmDAR2b | GRMZM2G151934_T01 |
| ZmLIM | GRMZM2G385236_T01 |
| ZmLIM3 | GRMZM2G017845_T01 |
| ZmLIM5 | GRMZM2G099328_T02 |
| ZmLIM9 | GRMZM2G153268_T01 |
| ZmLIM12 | GRMZM2G175761_T01 |
| ZmTAG-273 | GRMZM2G010960_T01 |
| AtWLIM1 | AT1G10200.1 |
| AtWLIM2A | AT2G39900.1 |
| At WLIM2B | AT3G55770.7 |
| AtPLIM2A | AT2G45800.1 |
| AtPLIM2B | AT1G01780 |
| AtPLIM2C | AT3G61230 |
| AtDAR1 | AT4G36860 |
| AtDAR3 | AT5G66640 |
| AtDAR4 | AT5G17890 |
| AtDAR5 | AT5G66630 |
