# Supplementary material for: Genome-Wide Analysis of LIM Family Genes in Foxtail Millet (Setaria italica L.) and Characterization of the Role of SiWLIM2b in Drought Tolerance
Source: Int J Mol Sci. 2019 Mar 15;20(6):1303. doi: 10.3390/ijms20061303 (PMC6470693; doi:10.3390/ijms20061303)
Supplement: Supplementary file 1 [file ijms-20-01303-s001.zip › ijms-442293-Supplemental/Supplemental Table S5.docx]

**Supplementary Table S5. Expression Profile Data of Different Tissues of *LIMs* in Foxtail Millet**

| **Locus Name** | **Log_2_RPKM** | | | |
| --- | --- | --- | --- | --- |
|  | **leaf** | **root** | **stem** | **tassel inflorescence** |
| **Seita.9G201000.1** | **3.584963** | **2** | **3** | **2.584963** |
| **Seita.9G164800.1** | **4.807355** | **5.83289** | **6.066089** | **5** |
| **Seita.3G375500.1** | **5.807355** | **5.285402** | **5.754888** | **4.459432** |
| **Seita.9G459200.1** | **3** | **4.643856** | **4.247928** | **4.087463** |
| **Seita.4G050800.1** | **3** | **4.169925** | **3.807355** | **3.807355** |
| **Seita.1G250500.1** | **0** | **0** | **NA** | **2** |
| **Seita.4G104700.1** | **2.584963** | **3.807355** | **4.754888** | **3.584963** |
| **Seita.3G353200.1** | **NA** | **6.882643** | **5.392317** | **5.70044** |
| **Seita.7G179500.1** | **3.459432** | **1.584963** | **2.321928** | **2.321928** |
| **Seita.9G458000.1** | **4** | **7.599913** | **6.870365** | **7.807355** |
