# Supplementary figures and images for: Genome-Wide Analysis of LIM Family Genes in Foxtail Millet (Setaria italica L.) and Characterization of the Role of SiWLIM2b in Drought Tolerance
Source: Int J Mol Sci. 2019 Mar 15;20(6):1303. doi: 10.3390/ijms20061303 (PMC6470693; doi:10.3390/ijms20061303)

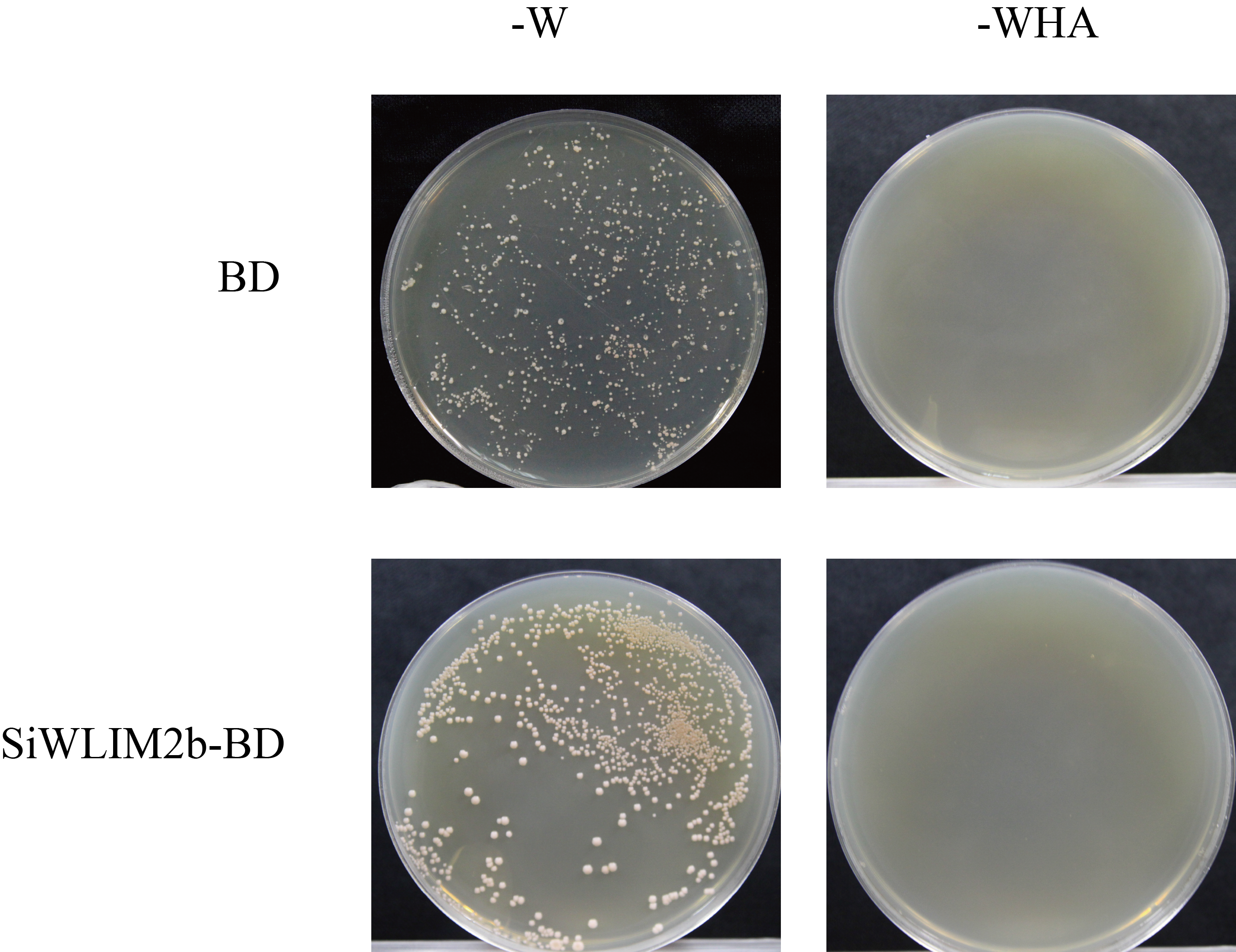

Supplement: Supplementary file 1 [file ijms-20-01303-s001.zip › ijms-442293-Supplemental/Supplemental Figure S1.png]
